# FLASH irradiation induces lower levels of DNA damage ex vivo, an effect modulated by oxygen tension, dose, and dose rate

**DOI:** 10.1259/bjr.20211150

**Published:** 2022-02-16

**Authors:** Christian R Cooper, Donald Jones, George DD Jones, Kristoffer Petersson

**Affiliations:** Leicester Cancer Research Centre, University of Leicester, Robert Kilpatrick Clinical Sciences Building, Leicester Royal Infirmary, Leicester, UK; Leicester Cancer Research Centre, University of Leicester, Robert Kilpatrick Clinical Sciences Building, Leicester Royal Infirmary, Leicester, UK; Leicester Cancer Research Centre, University of Leicester, Robert Kilpatrick Clinical Sciences Building, Leicester Royal Infirmary, Leicester, UK; MRC Oxford Institute for Radiation Oncology, University of Oxford, Old Road Campus Research Building, Oxford, UK; Department of Haematology, Oncology and Radiation Physics, Skåne University Hospital Lund University, Lund, Sweden

## Abstract

**Objective:**

FLASH irradiation reportedly produces less normal tissue toxicity, while maintaining tumour response. To investigate oxygen’s role in the ‘FLASH effect’, we assessed DNA damage levels following irradiation at different oxygen tensions, doses and dose rates.

**Methods:**

Samples of whole blood were irradiated (20 Gy) at various oxygen tensions (0.25–21%) with 6 MeV electrons at dose rates of either 2 kGy/s (FLASH) or 0.1 Gy/s (CONV), and subsequently with various doses (0–40 Gy) and intermediate dose rates (0.3–1000 Gy/s). DNA damage of peripheral blood lymphocytes (PBL) were assessed by the alkaline comet assay.

**Results:**

Following 20 Gy irradiation, lower levels of DNA damage were induced for FLASH, the difference being significant at 0.25% (*p* < 0.05) and 0.5% O_2_ (*p* < 0.01). The differential in DNA damage at 0.5% O_2_ was found to increase with total dose and dose rate, becoming significant for doses ≥20 Gy and dose rates ≥30 Gy/s.

**Conclusion:**

This study shows, using the alkaline comet assay, that lower levels of DNA damage are induced following FLASH irradiation, an effect that is modulated by the oxygen tension, and increases with the total dose and dose rate of irradiation, indicating that an oxygen related mechanism, *e.g.* transient radiation-induced oxygen depletion, may contribute to the tissue sparing effect of FLASH irradiation.

**Advances in knowledge:**

This paper is first to directly show that FLASH-induced DNA damage is modulated by oxygen tension, total dose and dose rate, with FLASH inducing significantly lower levels of DNA damage for doses ≥20 Gy and dose rates ≥30 Gy/s, at 0.5% O_2_.

## Introduction

Despite important technological advances in dose delivery precision, using image-guided and intensity-modulated radiotherapy, radiation therapy still induces irreversible side-effects.^1^ A key restraint on curative cancer care arises from normal tissue toxicity, limiting the total dose a tumour can receive.^2^ Current radiation research is focused on developing new treatment modalities, with the aim to reduce the risk of complications arising from radiation treatment, including inducing secondary tumours.^3,4^

FLASH radiotherapy (FLASH RT) is a new treatment modality that delivers radiation in a fraction of a second at ultra-high dose rates (≥40 Gy/s), which could revolutionise the field by improving the cancer therapeutic ratio,^2^ with heightened recent interest long after pioneering studies of over 40 years ago.^5,6^ FLASH RT has been observed to create a differential ‘FLASH effect’, sparing normal tissue while maintaining antitumour efficacy equal to that of conventional dose rate radiotherapy (CONV RT), in mice,^7–10^ minipigs and cats.^11^ More recently, a study found electron FLASH RT (430–500 Gy/s) as a suitable single fraction treatment alternative for the treatment of canine tumours at low tissue depths (2–3 cm), using a 10 MeV electron beam.^12^ In addition to clinical veterinary studies, the first patient (CD30+ T cell cutaneous lymphoma) was treated successfully with FLASH in 2018.^11^ However, the mechanisms underpinning the normal tissue sparing properties seen following irradiation at ultra-high dose rates have yet to be elucidated.^13^

It has been proposed that FLASH, due to its extremely short ultra-high dose rate delivery of radiation, transiently consumes local oxygen. This in turn better enables the thiol-(RSH)-mediated chemical ‘repair’ of radiation-induced secondary and tertiary organic radicals, so competing with oxygen’s ‘fixation’ of damage.^14–20^ Consequently, one likely outcome of FLASH exposure is lower levels of radiation-induced damage formation, including DNA strand breakage, with this contributing to the observed normal tissue sparing effect. Conversely, and in the context of improved therapeutic ratio, tumour tissues that are already hypoxic may not be protected as much from any oxygen consumption/depletion that FLASH induces.^2,8^ However, this remains to be experimentally established.

A reduction in cell death *in vitro* as well as a reduction in neurocognitive damage of irradiated mice *in vivo*, both dependent on oxygen concentration, have been reported for FLASH compared to CONV RT at the same total dose.^8,19^ Furthermore, a recent *in vivo* study by Cao^21^ and co-workers utilised phosphorescence quenching of a water-soluble molecular probe to measure oxygen consumption per unit dose using a 10 MeV electron beam. They identified oxygen depletion as higher in normal tissues (≈2.5 mmHg) than in tumours (≈1.02 mmHg) when irradiating mice with 20 Gy total dose, at ultra-high dose rates (90, 180 and 270 Gy/s); while irradiation of mice with CONV resulted in no change in partial pressures of oxygen. They also carried out oxygen consumption measurements in aqueous solution and found that CONV depletes higher amounts of oxygen (0.19–0.21 mmHg/Gy) than irradiation at ultra-high dose rates (0.16–0.17 mmHg/Gy). However, radiolytic oxygen depletion of a solution in a closed system does not adequately reflect the mechanisms of cellular oxygen depletion and reoxygenation during FLASH and CONV irradiation.^22,23^

Further *in-vitro* studies of FLASH radiolytic oxygen depletion by Khan *et al*.^24^ have shown that the hypoxic core of A549 spheroids may expand under FLASH RT (90 Gy/s) engulfing a large number of well-oxygenated cells, while oxygen is steadily replenished during CONV. They found clonogenic survival to be threefold higher in FLASH irradiated spheroids compared to CONV irradiation; with no difference found in two-dimensional well-oxygenated cultured cells, corroborating earlier studies outlining oxygen as a key determinant of the FLASH effect.^8,19,25,26^

In the present study, our aim is to investigate any potential differential in DNA damage in whole blood peripheral blood lymphocytes (PBL), using the alkaline comet assay, following *ex-vivo* irradiation at FLASH or CONV dose rates, and how this differential effect may be modified by oxygen concentration, total irradiation dose and dose rate.

## Methods and materials

The alkaline version of the comet assay [also known as single-cell gel electrophoresis (SCGE)] was performed to assess DNA damage formation in human PBL in fresh whole blood, taken from a single healthy donor, following *ex-vivo* exposure to FLASH or CONV irradiation. PBLs were chosen primarily for convenience (negating need for cell culture plus the problems/concerns of transporting cultures, and suitable volumes of whole blood samples can be readily obtained by finger pin-prick immediately onsite prior to Comet sample preparation), but do represent a body-wide, systemic normal tissue susceptible to radiation exposure.^27–29^ The alkaline version of the comet assay is the most sensitive version of the assay detecting both DNA strand breaks and alkaline-labile sites, with the whole blood PBL being irradiated embedded in thin low melting point agarose gels on glass microscope slides. The agarose gels were prepared by mixing 5 µl whole blood containing EDTA (0.16 mg/100 µl) with 190 µl of 37°C low melting point agarose, with 2 × 80 µl of this mix being used to prepare one gel on two separate slides. For further details of the alkaline comet assay procedure, see Supplementary Material 1.

### Varying oxygen concentration

Following slide preparation, the slides were placed in a HypoxyLAB (Oxford Optronix Ltd., Oxford, UK) hypoxia chamber for 2 h set to ≥85% humidity, 20°C. This was performed for five different partial pressures of oxygen (pO_2_) settings of the chamber; 0.25, 0.5, 0.75, 1 and 3% (2, 4, 6, 8 and 24 mmHg). These setting were varied prior to samples being placed in the chamber through flooding of N_2_ into the chamber until the desired pO_2_ was reached, which was controlled by the built-in O_2_ sensor (calibration and functionality regularly tested through measurements with an Oxylite sensor, Oxford Optronix Ltd.). At the end of the 2 h incubation, slides were placed inside 50 ml centrifuge tubes that had been stored at low oxygen tension overnight prior to use, and tightly sealed with the lid wrapped in nescofilm inside the chamber.

The tubes containing the slides were positioned so that the slide-mounted blood/agarose gels were perpendicular to and facing the beam, then irradiated at room temperature at FLASH (2 kGy/s) or CONV (0.1 Gy/s) dose rates to 20 Gy total dose. 5 min following start of irradiation, the tubes were opened and slides placed into ice cold lysis buffer. Slides irradiated under normoxia (21% O_2_) were similarly prepared and irradiated in 50 ml centrifuge tubes left at atmospheric O_2_ then placed into lysis. Unirradiated 21% O_2_ controls were placed into lysis solution immediately following their preparation plus the time of mock-irradiation to lysis (5 min). Prepared unirradiated 0.5% O_2_ equilibrated control slides were placed into lysis after all other slides had been irradiated.

### Varying irradiation dose

A similar procedure was used to evaluate whether a differential in DNA damage between FLASH (1.5 MGy/s-1.7 kGy/s for a 5–40 Gy delivery) *vs* CONV (0.1 Gy/s) was modulated by total dose. Slides equilibrated at 0.5% O_2_ (prepared as described above) were irradiated to a total dose of 5–40 Gy. Slides were placed into ice cold lysis buffer 5 min following start of irradiation (5–20 Gy) or 9 min following start of irradiation (30 and 40 Gy), in order to accommodate the protracted CONV irradiation times. Preliminary studies revealed that the whole blood PBLs used were devoid of any significant repair at room temperature over 40 min.

### Varying dose rates

PBL DNA damage levels was also investigated for a variety of intermediate dose rates, ranging from 0.3 Gy/s to 1 kGy/s. Again, slides equilibrated at 0.5% O_2_ (prepared as described above) were irradiated to a total dose of 20 Gy and placed into ice cold lysis buffer 5 min following the start of irradiation.

### Beam characteristics

**Figure 1.**
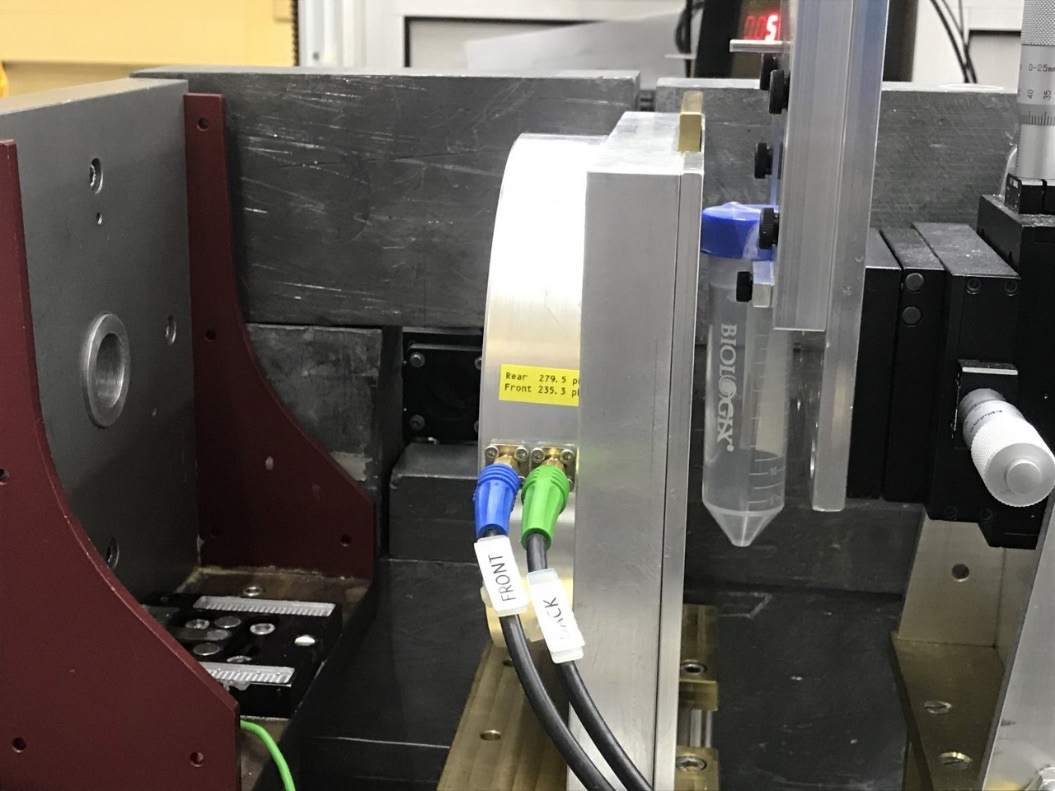
Image of the irradiation setup: each slide was sealed inside a centrifuge tube prior to irradiation (see Materials and Method), with the glass slide-mounted agarose gel containing whole blood sample centred against the collimation system perpendicular to the electron beam.

All irradiations were performed with a FLASH‐optimized in‐house developed linear accelerator (linac), which has been described in more detail elsewhere,^30^ delivering electrons of 6 MeV nominal energy with a circular horizontal beam of 5 cm in diameter, with each of the tubes containing the slides placed in contact with the collimation system so that the blood/agarose gels were centred in and perpendicular to the beam (Figure 1).

#### FLASH irradiation

All FLASH irradiations were performed with pulses of 5 Gy, each with a duration of 3.4 µs (pulse width), a pulse dose rate (or instantaneous dose rate) of 1.5 MGy/s and a pulse repetition frequency of 300 Hz, *e.g.* 4 pulses were used for a 20 Gy delivery, for a total irradiation time of 0.01 s and an average dose rate of 2 kGy/s.

#### Conventional irradiation

For conventional irradiation, pulses of 4 mGy were used, each with a duration of 3.4 µs (pulse width), a pulse dose rate (or instantaneous dose rate) of 1.2 kGy/s, a pulse repetition frequency of 25 Hz, and an average dose rate of 0.1 Gy/s, *e.g.* a 20 Gy delivery had a total irradiation time of 200 s.

#### Intermediate dose rates

For the eight intermediate dose rates levels, the amplitude of the 3.4 µs electron pulses were controlled by varying the electron gun current on the linac. Also, the pulse repetition frequency was set to 300 Hz for the 5 higher dose rates and at 25 Hz for the three lower dose rates, in order to achieve the desired average dose rates (Table 1).

**Table 1.** Irradiation parameters for a 20 Gy delivery at varying average dose rates

Average dose rate (Gy/s)	Total irradiation time (s)	Pulse repetition frequency (Hz)	Number of pulses (n)	Dose-per-pulse (Gy)	Pulse dose rate (kGy/s)
0.1	200	25	5001	0.004	1.2
0.3	67	25	1668	0.012	3.5
1	20	25	501	0.04	12
3	6.7	25	168	0.12	35
10	2	300	601	0.033	98
30	0.67	300	201	0.1	29
100	0.2	300	61	0.33	96
300	0.067	300	21	0.95	280
1000	0.02	300	7	2.9	840
2000	0.01	300	4	5	1500

### Dosimetry procedure

Preceding and following irradiation of each experiment and change in beam settings, radiochromic film (GafChromic EBT-XD, Ashland Inc., Covington, KY) was irradiated to verify the dose delivered. Pieces of film (3.3 × 2.3 cm^2^) were positioned directly on a microscope slide which was placed inside a 50 ml centrifuge tube and positioned as the blood/agarose samples in (and perpendicular to) the beam and irradiated with the defined beam settings. The films were scanned 24 h post-irradiation (Epson Perfection v850 Pro, Seiko Epson Corporation, Nagano, Japan) and the red channel analysed (ImageJ v. 1.52a, Wayne Rasband, NIH). The averaged dose over a 1.2 × 2.0 cm^2^ central part of the film was recorded. The films had previously been calibrated in a 6 MeV clinical electron beam from a Varian Truebeam linac (Varian Medical Systems Inc., Palo Alto, CA). For online verification of the dose delivery, a toroidal beam charge monitor as well as a beam energy monitor was used.^30^ The energy monitor was also used to verify that the electron beam energy was consistently 6 MeV. Our overall uncertainty in dosimetry was estimated to be 4%, including a measured output variation of our FLASH and CONV IR deliveries of within 2%. For intermediate dose rates, due to an increased output variation, the overall uncertainty in dosimetry was estimated to be 8%.

### Electrophoresis & comet visualisation/Scoring

The following steps were carried out in the dark, under red light. On removal from lysis, slides were washed twice for 10 min with double distilled water (ddH_2_O) and incubated in ice-cold electrophoresis buffer for 20 min. Electrophoresis was then performed for 20 min at 30 V (0.8 V/cm) 300 mA. Slides were then incubated in neutralisation buffer for 20 min, washed two times with ddH_2_O and allowed to dry at 37°C.

Once dry, slides were rehydrated with ddH_2_O for 20 min then stained with approximately 1 ml propidium iodide (PI) diluted in ddH_2_O (2.5 µg ml^−1^) for 25 min, washed two times with ddH_2_O and dried at 37°C. Comets were visualized at X20 magnification using a fluorescence microscope (Zeiss Axioskop 450; Carl Zeiss, Jena, Germany) fitted with an excitation filter of 515–535 nm, a barrier filter of 590 nm and a 100 W mercury lamp.

Images were captured from the microscope using an attached Stingray F-046B digital camera (Allied Vision Technologies, Stadtroda, Germany) connected to a computer running COMET IV software (Perceptive Instruments, Instem, Cambridge, UK). Comets were captured and scored by randomly selecting 50 comets from the centre of each gel.

### Statistical analysis

COMET IV Software calculated % Tail DNA automatically, producing a spreadsheet of data for each slide; % Tail DNA was selected for use as the Comet parameter that best reflects DNA damage.^31^ Each individual experiment had up to three internal replicates (constituting three slides = three gels) and each experiment was repeated three times to yield the mean and standard deviation of each test condition. Graphs were created using GraphPad Prism 9 (GraphPad Software, La Jolla, CA).

Statistical analysis was performed using the ‘Analyze’ tool on GraphPad Prism. A two-tailed unpaired *t*-test was used to assess statistically significant differences (*p* < 0.05) between % Tail DNA values (FLASH *vs* CONV) at different oxygen concentrations, at different total doses and various dose rates.

## Results

### Varying oxygen concentration

**Figure 2.**
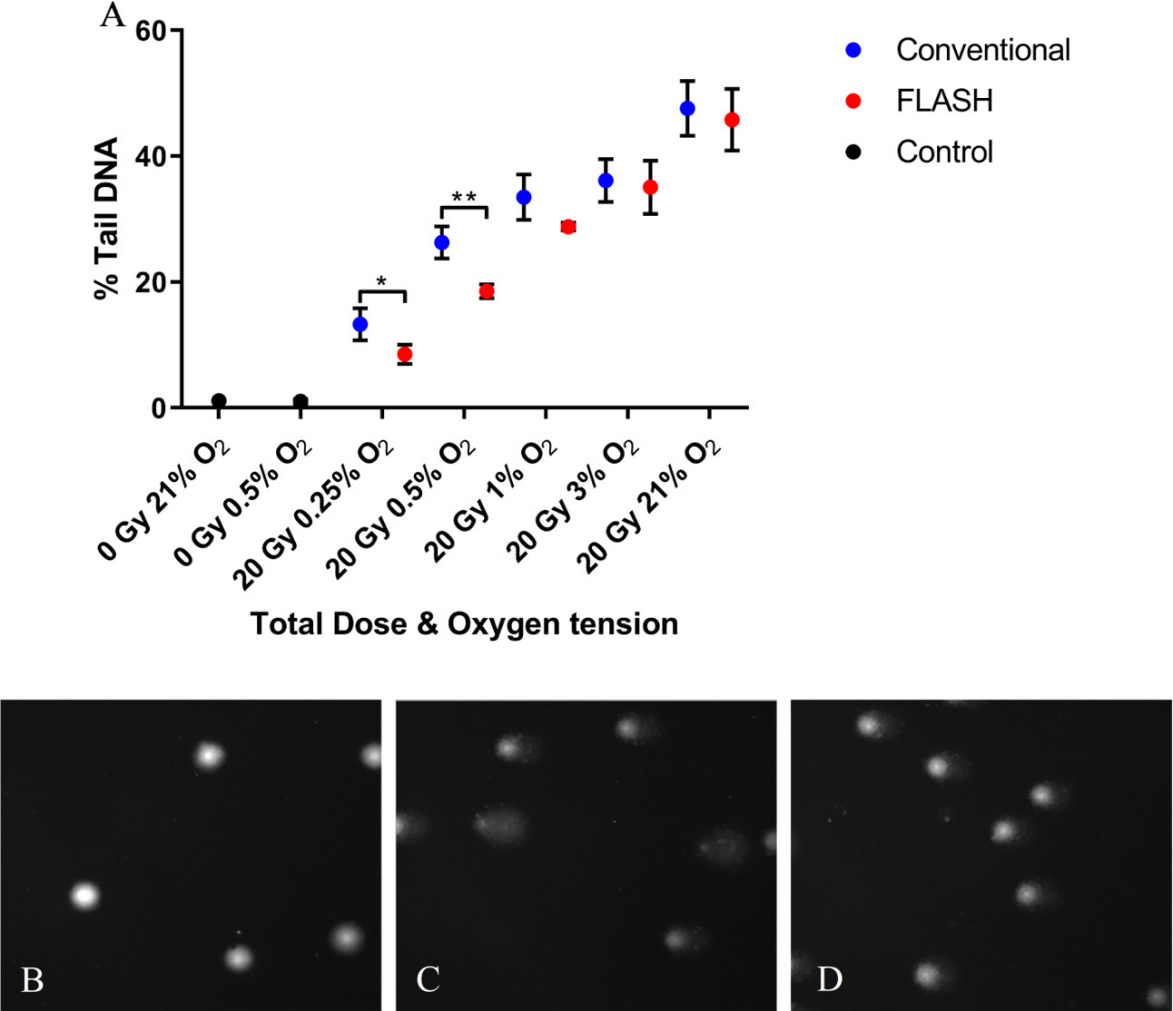
**A**) Alkaline comet assay measures of peripheral blood lymphocytes (PBL) DNA damage formation (% Tail DNA) following 20 Gy FLASH (2 kGy/s) or conventional dose rate (CONV, 0.1 Gy/s) irradiation over 0.25–21% oxygen tension. Data expressed as the mean % Tail DNA of three slides (*n* = 3); error bars indicate standard deviation of the means for each experimental condition. Statistical analysis (two-tailed unpaired *t*-test) FLASH *vs* CONV revealed significant differences (*, *p* < 0.05) at 0.25% O_2_ and (**, *p* < 0.01) at 0.5% O_2_. Comet images captured of B) PBL unirradiated at 0.5% O_2_; **C**) PBL 20 Gy CONV irradiated at 0.5% O_2_; **D**) PBL 20 Gy FLASH irradiated at 0.5% O_2_. PBL, peripheral blood lymphocyte.

A small (non-significant) difference in DNA damage of PBL was noted following irradiation (20 Gy total dose) when delivered at FLASH (2 kGy/s) *vs* CONV (0.1 Gy/s) dose rates at 21% O_2_ (Figure 2), with FLASH inducing a 2% Tail DNA (mean) value lower than CONV. Similarly, a small (non-significant) difference of 1% Tail DNA was observed between FLASH and CONV at 3% O_2_. At 1% O_2_, a larger difference (though non-significant) of 5% Tail DNA was observed between FLASH *vs* CONV irradiation (Figure 2).

However, significant differences of 8 and 5% Tail DNA were observed between FLASH and CONV at 0.5% O_2_ (**, *p* < 0.01) and at 0.25% (*, *p* < 0.05) (Figure 2). These respective % Tail DNA values correspond to relative DNA damage differences between FLASH and CONV (mean values of %Tail DNA, $\frac{FLASH-control}{CONV-control}$ ) of 0.96, 0.97, 0.85, 0.69, and 0.61 for 21%, 3%, 1%, 0.5%, and 0.25% O_2_, respectively.

### Varying dose

**Figure 3.**
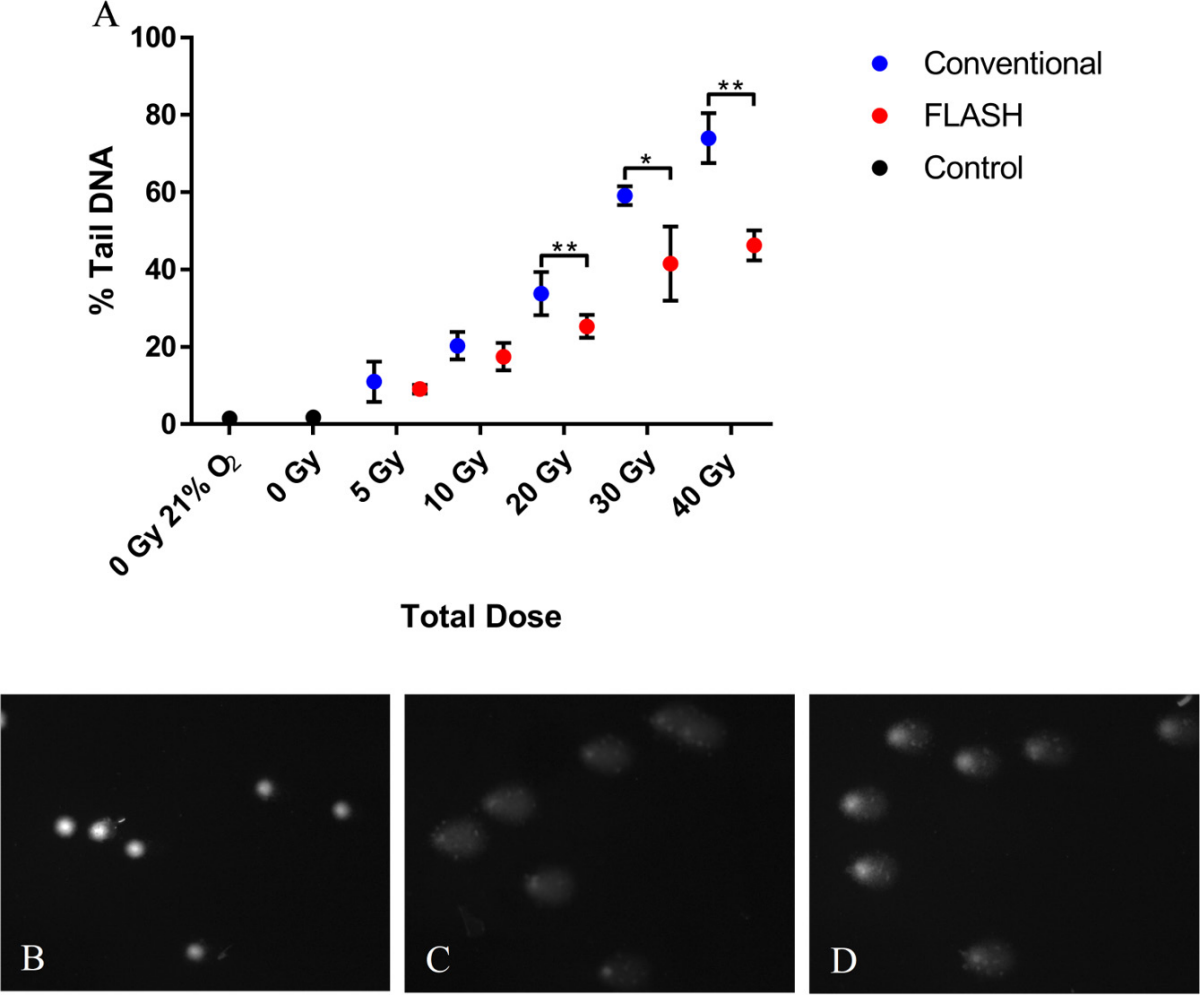
**A**) Alkaline comet assay measures of PBL DNA damage formation (% Tail DNA) following FLASH (2 kGy/s) or conventional dose rate (CONV, 0.1 Gy/s) irradiation to various total doses. Data expressed as the mean % Tail DNA of three slides (*n* = 3); error bars indicate standard deviation of the means for each experimental condition. Statistical analysis (two-tailed unpaired *t*-test) FLASH *vs* CONV revealed no significant differences for doses ≤ 10 Gy. However, statistically significant differences were found for 20, 30 and 40 Gy (**, *p* < 0.01; *, *p* < 0.05). Comet images of B) PBL unirradiated 0.5% O_2_; **C**) PBL 40 Gy CONV irradiated at 0.5% O_2_; **D**) PBL 40 Gy FLASH irradiated at 0.5% O_2_. PBL, peripheral blood lymphocyte.

Again, the same difference in PBL DNA damage (8% less Tail DNA) between FLASH and CONV irradiation was observed following 20 Gy irradiation at 0.5% O_2_ (Figure 3). The difference in DNA damage between FLASH and CONV was seen to increase with total dose delivered. For 5 Gy FLASH, the difference in % Tail DNA (mean value) between FLASH and CONV was 2%. The difference increased with dose to 3% for 10 Gy, and became significant at 20, 30 and 40 Gy with differences of 8%, 18% and 28%, respectively (Figure 3). This corresponds to a relative DNA damage difference between FLASH and CONV (mean values of %Tail DNA, $\frac{FLASH-control}{CONV-control}$ ) of 0.79, 0.85, 0.73, 0.69 and 0.62 for 5, 10, 20, 30 and 40 Gy, respectively.

### Varying dose rates

**Figure 4.**
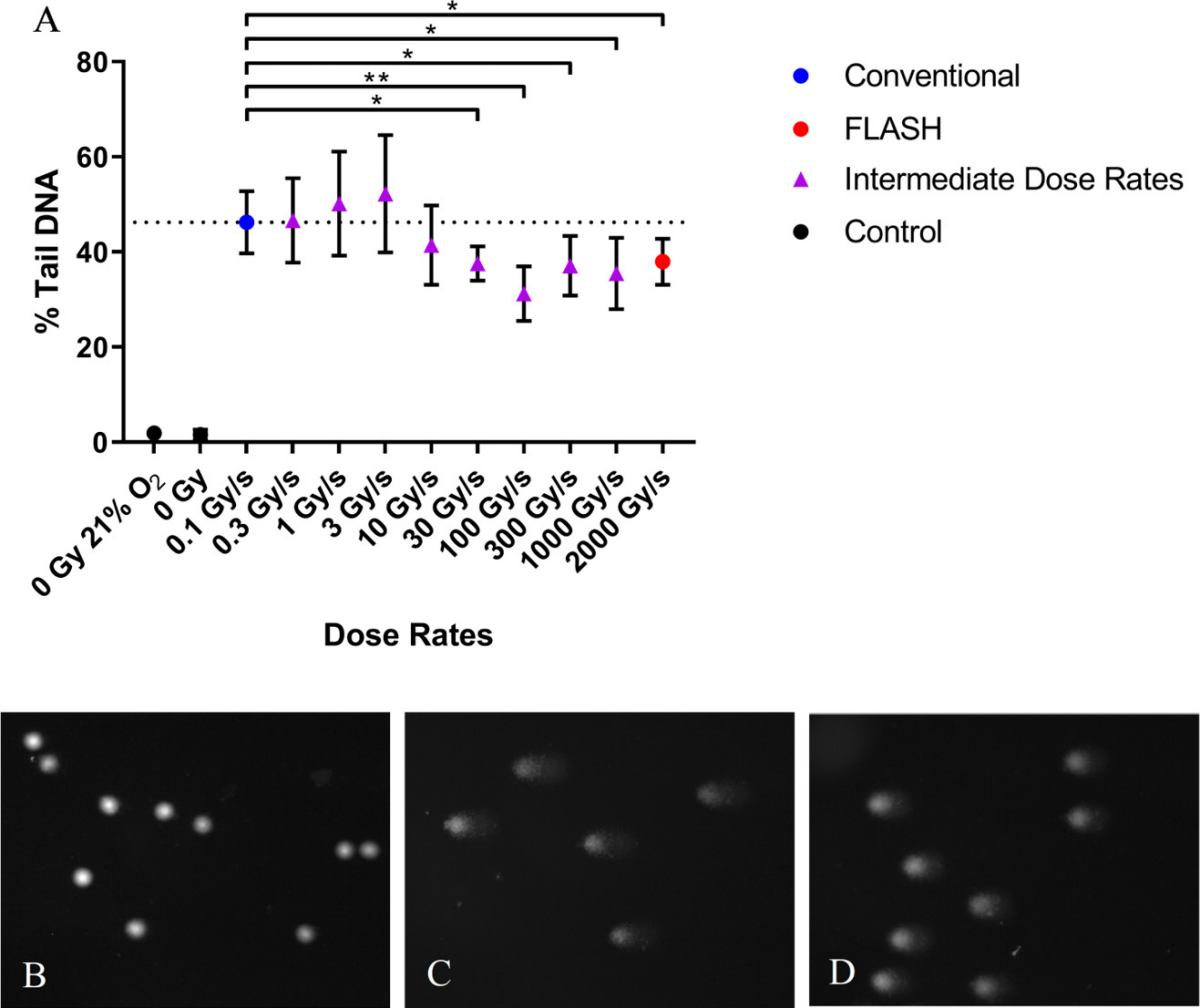
**A**) Alkaline comet assay measures of PBL DNA damage formation (% Tail DNA) irradiated to 20 Gy total dose at various dose rates 0.1–2kGy/s. Data expressed as the mean % Tail DNA of three slides (*n* = 3); error bars indicate standard deviation of the means for each experimental condition. Horizontal dashed line represents % Tail DNA value for 0.1 Gy/s. Statistical analysis (two-tailed unpaired *t*-test) FLASH *vs* CONV revealed no significant differences for dose rates ≤ 10 Gy/s. However, statistically significant differences were found between 0.1 Gy/s and dose rates ≥ 30 Gy/s (**, *p* < 0.01; *, *p* < 0.05). Comet images of B) PBL unirradiated at 0.5% O_2_; **C**) PBL 0.1 Gy/s CONV irradiated at 0.5% O_2_; **D**) PBL 100 Gy/s FLASH irradiated at 0.5% O_2_. PBL, peripheral blood lymphocytes.

A difference in DNA damage formation (% Tail DNA, mean values) was found for samples exposed to 20 Gy irradiation at CONV dose rate (0.1 Gy/s) and samples exposed to dose rates ≥10 Gy/s, which became significant for dose rates ≥30 Gy/s (Figure 4). Differences in % Tail DNA of 5%, 9%, 15%, 9%, 11% and 8% were found between 0.1 Gy/s and 10, 30, 100, 300, 1000, and 2000 Gy/s, respectively (Figure 4). This corresponds to relative DNA damage differences (mean value of %Tail DNA, $\frac{\left[10-2000Gy/s\right]-control}{CONV-control}$ ) of 0.83, 0.81, 0.66, 0.79, 0.76 and 0.81 for 10, 30, 100, 300, 1000 and 2000 Gy/s, respectively.

## Discussion

To date, studies of the FLASH effect and notably the proposed radiolytic consumption of oxygen as a driver of the FLASH sparing of normal tissues, have largely investigated models utilizing water.^32,33^ In these studies, it is proposed that the products of water radiolysis (namely hydrated electrons (e*_aq_* ⁻) and hydrogen radicals (H•)) react with the dissolved molecular oxygen to produce superoxide anions and perhydroxyl radicals, respectively. However, such reactions will not occur to any significant extent within cells because of the high concentrations of competing scavengers, and such studies have attracted criticism as being inappropriate for the study of FLASH *vs* CONV exposures.^34,35^ A more credible route for the radiolytic consumption of oxygen is via oxygen reacting with radiation-induced secondary and tertiary organic radicals that will occur on the millisecond-or-greater timescale.^35^ Therefore, further *in vitro* work, enabling superior parameter variation and control *vs in-vivo* studies, is warranted to uncover the likely mechanisms underpinning the benefits of FLASH. This formed the rationale of our *ex-vivo* irradiations (FLASH *vs* CONV), varying oxygen concentration, dose, and dose rate to corroborate previous cell survival and *in-vivo* studies.^8,10,19,36^

The alkaline comet assay was chosen as a suitable method to assess DNA damage formation in this study, as it allows for the direct analysis of PBL DNA damage induced following FLASH and CONV irradiation.^37^ The assay uses gel electrophoresis of lysed cell nucleoids combined with fluorescence microscopy to allow for the assessment of broken DNA from individual cells.^38^ The alkaline version of the assay detects single and double strand breaks and breaks resulting from alkaline labile sites,^39^ which were relatively quantified as Tail DNA (%).^31^

The comet assay shows a small non-significant sparing of DNA damage for FLASH *vs* CONV irradiation at 21% O_2_. A similar non-significant sparing has previously been shown for FLASH in studies of clonogenic survival at 21%, which grows to become significant at lower oxygen concentrations (1–4%), similar to the comet assay data.^19,23,40^ Also at 21% O_2_, Fouillade *et al.* found that FLASH induces less initial, 53BP1-relevant DNA damage than CONV and that this difference is specific to normal cells,^41^ while Adrian *et al.* found no significant difference in 53BP1-relevant DNA damage but a difference in clonogenic survival that was cell line dependent.^42^ In contrast, Buonanno *et al.* found no statistical difference in clonogenic survival for proton FLASH *vs* CONV but found a saturation of γH2AX foci formation beyond 10 Gy for the highest dose rate used (1000 Gy/s), an effect that was not seen for lower dose rates (0.05 and 100 Gy/s).^43^ Similarly, the comet assay shows sparing of DNA damage for FLASH above 10 Gy, albeit at a lower oxygen concentration of 0.5%.

Transient hypoxia due to radiolytic oxygen depletion following FLASH may account for the significant differential in PBL DNA damage (% Tail DNA) seen at 0.5 and 0.25% O_2_ *vs* CONV irradiation (20 Gy total dose delivered), with this sparing effect being modulated by varying the oxygen concentration (Figure 2). When DNA radicals are produced from ionizing radiation, they may react with oxygen to form peroxyl radicals yielding higher levels of permanent DNA damage; but with the caveat that both peroxyl formation and thiol repair may be reversible/not permanent.^15,16,44^ Previous studies have measured radiolytic oxygen depletion in aqueous solutions to be around 0.20 mmHg/Gy (*i.e.* 0.5% or 4 mmHg/20 Gy), which suggests that the 0.5 and 0.25% O_2_ local oxygen is likely fully consumed in the blood/agarose samples during a 20 Gy delivery,^21,23^ through reaction with radiation-induced secondary and tertiary organic radicals. Subsequently, the DNA radicals produced under hypoxia may better undergo thiol mediated chemical repair leading to lower levels of strand break damage formation.^14–20^ However, due to the more protracted delivery time for CONV, oxygen is better replenished inside the cell and the oxic conditions maintained. The results from this study meet the expectation that FLASH may induce a local transient hypoxia that may not be visible above or below a certain threshold of oxygen.^18,19,24,45,46^ In relation to the results seen in Figure 2, it has been proposed that normal tissue protection following FLASH irradiation *in vivo* may arise via sparing of hypoxic stem cell niches.^47^ These have the potential to elicit a hypoxic response to FLASH irradiation, via oxygen depletion, which may account for conferred radioresistance causing greater stem cell sparing under FLASH *vs* CONV irradiation.

Increasing irradiation dose rates to ultra-high levels may provide a sparing effect in terms of reducing DNA damage in normal tissues.^10,41^ In our study, this was observed in healthy human PBL for dose rates of ≥10 Gy/s, which became significant for dose rates ≥ 30 Gy/s (Figure 4). This is similar to the neurocognitive sparing seen in the mice study by Montay-Gruel *et al*.^36^ Likewise, increasing the total dose delivered to PBL at 0.5% O_2_ (Figure 3) was also found to create a greater differential between FLASH and CONV DNA damage values (≥20 Gy). Subjecting PBL to ≥20 Gy total dose at 0.5% O_2_ will sufficiently deplete oxygen, enabling less DNA damage formation under FLASH irradiation in comparison to CONV dose rates (see above).

According to our results, radiolytic oxygen depletion, leading to transient hypoxia may be a contributor for the FLASH survival/sparing effect seen *in vitro*.^8,19,40^ However, radical–radical reactions may also contribute.^34,48^ Furthermore, a small sparing effect has also been observed for some cell lines that appears to be unrelated to the oxygen content and thereby not explained by oxygen depletion.^41,42^ A differential in immune response^49^ (possibly as a consequence of hypoxia) may also contribute to the FLASH sparing effect seen *in vivo*. Furthermore, considering our data, the FLASH sparing effect seen *in vivo* in normal tissues at higher oxygen tensions, may only in part be driven by oxygen depletion. It is imperative to truly understand the role of radiolytic oxygen depletion and the ‘FLASH effect’, as to whether it may provide a sparing effect not just to normal tissues but also to solid tumour types that contain sufficient oxygen to produce a lowered radiosensitivity under FLASH irradiation.^19^

## Conclusions

We have identified an *ex-vivo* FLASH sparing effect. A differential in DNA damage formation of human PBL between CONV and FLASH irradiation is present at low oxygen tension, significantly at 0.25 and 0.5% O_2_. We have also shown that this differential in DNA damage is modulated by total dose and dose rate, finding it to increase with total dose delivered and resulting in significantly less DNA damage formation in PBL irradiated at dose rates ≥30 Gy/s in comparison to 0.1 Gy/s. Our findings of FLASH irradiation inducing lower levels of DNA damage meets an expectation of FLASH-induced transient oxygen depletion, and these two factors together (induced hypoxia and/or lower damage levels) may contribute to normal tissue sparing effects of FLASH radiotherapy *in vivo*.

## Supplementary Material

bjr.20211150.suppl-01
