# Supplementary material for: FLASH irradiation induces lower levels of DNA damage ex vivo, an effect modulated by oxygen tension, dose, and dose rate
Source: Br J Radiol. 2022 Feb 16;95(1133):20211150. doi: 10.1259/bjr.20211150 (PMC10993968; doi:10.1259/bjr.20211150)
Supplement: bjr.20211150.suppl-01 [file bjr.20211150.suppl-01.docx]

**Supplementary Material**

**Materials and Methods**

The steps of slide preparation, oxygen equilibration and irradiation were all undertaken at the MRC Oxford Institute for Radiation Oncology, University of Oxford, with the sample slides then being immediately transported to the Leicester Cancer Research Centre (LCRC), University of Leicester, in ice-cold lysis buffer (4.5M NaCl, 100mM Na_2_EDTA and 10mM Tris Hydrochloride in double distilled water (ddH_2_O) adjusted to pH 10, 1% Triton x-100 added fresh) on ice and held overnight at 4^o^C. The following day the SCGE protocol consisting of electrophoresis and comet visualisation/scoring was concluded at the LCRC.

Slide Preparation (at Oxford)

Double frosted end microscope slides were pre-coated with normal melting point agarose (1% prepared in ddH_2_O) and left to dry overnight at room temperature.

100 µl whole blood, taken from a single healthy volunteer by finger prick at the approximately same time on the day (ca. 9am), was mixed with 4 µl EDTA (0.16mg/100µl blood). A 5 µl aliquot of this blood-EDTA was mixed with 190 μl of molten low melting point agarose (0.6% in PBS) cooled to 37^o^C and immediately a 80 μl aliquot was taken and placed on the centre of the coated side of each slide. A coverslip was placed onto the droplet of blood/agarose (enabling even distribution of the gel) and the slide was placed onto a metal tray on ice. Slides were left on ice for 20 minutes, then the coverslips removed.

Electrophoresis & Comet Visualisation/Scoring (at Leicester)

The slides were removed from the lysis buffer and washed twice for 10 minutes with ice cold ddH_2_O. Slides were then transferred to the electrophoresis tank. Ice cold electrophoresis buffer (300mM NaOH and 1mM Na_2_EDTA in ddH_2_O, pH 13) was added to the tank and slides incubated for 20 minutes before electrophoresis was performed as described in the main text. Neutralisation buffer (0.4M Tris Base in ddH_2_O, pH 7.5) was used in the neutralisation step.

After staining with PI, the slides were stored in a light-tight box after drying prior to analysis. For comet visualisation, a drop of ddH_2_O was placed onto the centre of each slide/gel and the gel covered with a cover slip.
